# Genetic Characterization of MODY in Iranian Families Using Multigenerational‐Based Whole‐Exome Sequencing Approach

**DOI:** 10.1155/jdr/9383849

**Published:** 2025-12-30

**Authors:** Fateme Sefid, Masoud Dehghan Tezerjani, Samira Asadollahi, Seyed Ali Madani, Tayebeh Pardal, Maryam Imani, Zahra Salmani, Masoud Rahmanian, Hossein Hozhabri, Seyed Mehdi Kalantar, Mohammad Yahya Vahidi Mehrjardi

**Affiliations:** ^1^ Department of Medical Genetics, Shahid Sadoughi University of Medical Science, Yazd, Iran, ssu.ac.ir; ^2^ Research Center for Health Technology Assessment and Medical Informatics, School of Public Health, Shahid Sadoughi University of Medical Sciences, Yazd, Iran, ssu.ac.ir; ^3^ Diabetes Research Center, Non-Communicable Diseases Research Institute, Shahid Sadoughi University of Medical Sciences, Yazd, Iran, ssu.ac.ir; ^4^ Research Development Center, Shahid Sadoughi Hospital, School of Medicine, Shahid Sadoughi University of Medical Sciences, Yazd, Iran, ssu.ac.ir; ^5^ Reproductive Immunology Research Center, Shahid Sadoughi University of Medical Sciences, Yazd, Iran, ssu.ac.ir; ^6^ Department of Experimental Medicine, Sapienza University, Rome, Italy, uniroma1.it; ^7^ Abortion Research Center, Yazd Reproductive Sciences Institute, Shahid Sadoughi University of Medical Sciences, Yazd, Iran, ssu.ac.ir

**Keywords:** exome sequencing, GCK, HNF1A, MODY

## Abstract

**Background:**

Maturity‐onset diabetes of the young (MODY), a common type of monogenic diabetes caused by a pathogenic variant in a single gene, is characterized by starting at an early age, autosomal dominant inheritance, and decreased secretion of insulin. Despite its clinical importance, its accurate diagnosis is challenging, and it is often misdiagnosed as other types of diabetes. Therefore, understanding the genetic basis of MODY can improve diagnostic accuracy.

**Methods:**

We initially performed genetic counseling for 2964 probands who visited the Yazd Diabetes Center, Shahid Sadoughi University of Medical Sciences, Yazd, Iran, between 2018 and 2022. Clinical assessments and pedigree analyses were conducted for the accurate clinical diagnosis and management of diabetes. Among these, 11 probands with unknown types of diabetes who met specific criteria, including an inheritance pattern across at least three generations, at least seven affected individuals, and probands being under age 55, were selected for whole‐exome sequencing (WES). Finally, variants were verified by Sanger sequencing, pedigree analysis, and segregation analysis.

**Results:**

WES analysis detected pathogenic variants in two families, which confirmed MODY. Family 202 had a novel missense variant (GCK: c.484G > C; p.Gly162Arg; NM_000162.5). In Family 105, an extremely rare pathogenic frameshift variant (HNF1A: c.1136_1137del; p.Pro379ArgfsTer39; NM_000545.8) was identified. The segregation analyses of these variants also revealed that the variants largely co‐segregated with the diabetes phenotype in their respective families.

**Conclusion:**

This study clearly demonstrates the effectiveness of WES for the accurate identification of MODY subtypes in Iranian families. Moreover, these findings emphasize the need for further genetic screening programs in Iran to enhance MODY diagnosis, personalized treatment, and family genetic counseling.

## 1. Introduction

Monogenic diabetes (MD) includes maturity‐onset diabetes of the young (MODY), neonatal diabetes mellitus (NDM), and syndromic types of diabetes. Among these, MODY constitutes 1%–2% of the total diabetes prevalence in most studied populations [1]. The condition is characterized by early onset, at least two generations of autosomal dominant inheritance, and noninsulin dependence. The dysfunction of pancreatic beta cells in this disorder results in reduced glucose‐induced insulin production in early life, which is caused by a monogenic variant [2, 3].

Identification of individuals suffering from MODY is essential to ensure appropriate treatment and effective follow‐up, as well as genetic counseling for other family members. However, the misdiagnosis of MODY is a prevalent issue. It is estimated that approximately 5% of individuals with Type 2 diabetes mellitus (T2DM) and almost 10% of those with Type 1 diabetes mellitus (T1DM) may actually have MODY [4]. Moreover, around 80%–90% of individuals with MODY are not correctly diagnosed in some populations, such as the United Kingdom [5].

Advances in molecular genetics have resulted in the identification of causal variants in particular genes involved in *β*‐cell function. This has allowed the classification of MODY according to genetic etiology and the identification of various clinical phenotypes. These phenotypes differ in terms of characteristics such as the intensity of hyperglycemia, age of onset, response to therapy, prognosis, extra‐pancreatic abnormalities, and associated complications [6]. Notably, up to 80% of all cases of MODY are attributable to heterozygous variants in the *HNF1A/4A* and *GCK* genes [7]. Pathogenic variants in the HNF1A and HNF4A genes, encoding transcription factors hepatocyte nuclear factor‐1 alpha and 4 alpha, can lead to a progressive decline in insulin secretion and hyperglycemia. These changes may increase the risk of developing vascular complications related to diabetes. While sulfonylurea tablets are frequently used as an initial treatment for patients with pathogenic variants in these genes, insulin therapy may become necessary later in life [8, 9]. The intracellular enzyme glucokinase, which acts as a glucose sensor in pancreatic cells, is encoded by the GCK gene. Pathogenic variants in this gene result in a frequently asymptomatic, mild, and nonprogressive form of fasting hyperglycemia that generally does not require treatment, except in certain cases such as pregnancy [10, 11].

There are still reported cases of MODY that remain unexplained by genetics, even after extensive linkage analysis [12]. Furthermore, several studies have revealed that the frequency of particular MODY gene pathogenic variants varies significantly across various ethnic backgrounds and regions [6]. To date, the majority of studies have focused on identifying genetic causes of MODY in individuals of European descent, with only a limited number of investigations undertaken in Iran and in the Middle East [13–15]. Notably, the prevalence of MODY X (caused by unknown genes) is significantly higher in Asia, ranging from 80% to 90%. These findings suggest that Asians may carry additional, as yet unidentified, genetic variants implicated in the etiology of MODY [16–18]. In addition, given the increased prevalence of consanguineous marriages and the considerable burden of genetic homozygosity in the Middle East [19], further investigations are crucial for identifying MODY‐associated variants [20].

The final confirmation of MODY can only be made by finding pathogenic variants through whole‐exome sequencing (WES) and targeted gene sequencing. Therefore, extensive genome profiling studies are necessary in our country and region to precisely determine the number of cases and subgroups of MODY. In this study, we performed genotyping in individuals with unknown types of diabetes to identify any genetic abnormalities that could facilitate identification and reclassification into a type of MD, including MODY.

## 2. Material and Methods

### 2.1. Ethics

This study received approval from the Ethics Committee of Shahid Sadoughi University of Medical Sciences (ID: IR.SSU.REC.1396.216). This research pursued the guidelines of the Declaration of Helsinki, and all individuals provided informed consent prior to sample collection.

### 2.2. Experimental Subjects

We initially performed genetic counseling for 2964 probands who visited the Yazd Diabetes Center, Shahid Sadoughi University of Medical Sciences, Yazd, Iran, between 2018 and 2022 for the accurate diagnosis and management of diabetes. In total, these families included 13,748 individuals. The clinical diagnosis of diabetes was based on plasma glucose levels, either the HbA1C criteria or the fasting plasma glucose (FPG) and the 2‐h plasma glucose (2‐h PG) value following a 75‐g oral glucose tolerance test (OGTT), in compliance with the American Diabetes Association′s standards [21]. Following clinical assessment and genetic counseling, including pedigree analysis, families with unknown types of diabetes were selected for further investigation. Among these families, 103 pedigrees showed an observable inheritance pattern. Then, we selected the families from this group based on the following criteria: at least seven affected individuals; an observable inheritance pattern in at least three generations from either the paternal, maternal, or unilateral lineage; probands aged under 55 years at the time of evaluation; and the absence of any previously identified syndrome in the family linked to impaired glucose metabolism (e.g., Prader–Willi syndrome). These strict criteria were considered to increase the likelihood of identifying MD, including MODY, in families where the diabetes type remained unclear after standard clinical and biochemical evaluations. After applying these criteria, 11 probands were selected for molecular genetic analysis to determine the precise genetic subtype of diabetes.

### 2.3. WES

Genomic DNA (gDNA) was isolated from peripheral blood mononuclear cells of 11 probands and their affected relatives employing the Qiagen (United States) purification kit according to the manufacturer′s protocol. Next, DNA concentrations and quality were measured spectrophotometrically utilizing the NanoDrop 2000c (ThermoFisher Scientific, MA, United States). The isolated DNA was further evaluated using the Qubit dsDNA HS Assay Kit and the Qubit 3.0 Fluorometer (Invitrogen, CA, United States). For 11 probands, whole‐exome libraries were prepared with the TruSeq Exome Library Preparation Kit (Illumina, CA). Sequencing was carried out on the XTen system (Illumina, San Diego, CA, United States) that generated 2 × 150 bp paired‐end reads. The sequencing depth for each sample surpassed 100x.

### 2.4. WES Data Analysis

We initially assessed the quality of the raw data utilizing the FastQC (https://www.bioinformatics.babraham.ac.uk/projects/fastqc/) and NGS QC toolkits [21]. Subsequently, the data was filtered by eliminating adapters, contaminants, and poor‐quality reads. Reads with Phred quality score above 20 were considered high quality and aligned to the latest human reference genome (GRCh38) using the BWA tool (Burrows–Wheeler Aligner, v 0.7.17) [21]. The SAM (sequence alignment map) file was then converted to the Binary Alignment Map (BAM) format, and duplicate reads were identified and eliminated using the Picard tool(http://picard.sourceforge.net/). Then, we used GATK Base Quality Score Recalibration (BQSR) to perform base quality recalibration of the reads. The Integrative Genomics Viewer (IGV) software (Broad Institute, United States) was also used to evaluate the quality of sequence reads.

The variant calling process was carried out to identify single‐nucleotide variations (SNVs) and small insertions or deletions (indels) utilizing GATK HaplotypeCaller [22]. GATK Variant Quality Score Recalibration and ANNOVAR tools were used to remove false‐positive variants and for annotation, respectively [23]. Finally, three independent experts analyzed the annotated VCF to filter and prioritize the variants associated with phenotypes, considering the following criteria: (1) minor allele frequency (MAF) < 0.01 in population databases (gnomAD, 1000 Genomes, Genome Asia, and Iranome); (2) predicted functional impact (e.g., missense, nonsense, frameshift, or splice‐site variants); (3) segregation with phenotype; and (4) consistency with the inheritance pattern. The analysis was gene‐agnostic, and we did not limit our assessment to known MODY genes at the initial filtering stage. In addition, the Exomiser tool was employed in the filtering process [24]. A detailed summary of the variant filtering and prioritization steps for representative families is provided in Table S2.

### 2.5. In Silico Analysis

Multiple pathogenicity prediction tools, such as AlphaMissense (https://alphamissense.hegelab.org/), SIFT (https://sift.bii.a-star.edu.sg/sift4g/AnnotateVariants.html), MutationTaster (http://www.mutationtaster.org), and PolyPhen2 (http://genetics.bwh.harvard.edu/), were also employed to assess the effect of the variants on the protein sequence [25–27]. In addition, we assessed the pathogenicity of the identified variants based on the American College of Medical Genetics and Genomics (ACMG) criteria [28].

To predict and visualize the 3D protein structures, we used Google DeepMind′s AlphaFold. For the wild‐type GCK, we retrieved the precomputed structure available from the AlphaFold Protein Structure Database (https://alphafold.ebi.ac.uk/). For the wild type HNF1A and mutant forms (GCK: p.Gly162Arg and HNF1A: p.Pro379ArgfsTer39), we manually edited the protein sequences according to the identified genetic variants. The edited sequences were then input into a local implementation of the AlphaFold2 model in Google Colab (https://colab.research.google.com/github/sokrypton/ColabFold/blob/main/AlphaFold2.ipynb) to generate predicted structures. The per‐residue predicted local distance difference test (pLDDT) scores, which range from 0 to 100, were used to rate the model′s confidence: pLDDT > 90 means very high confidence (usually well‐modeled areas with accurate side‐chain placement); 70–90 means confident modeling (reliable backbone structure); 50–70 means low confidence (possible disorder or modeling uncertainty); and < 50 means very low confidence (often an unstructured or disordered region) [25].

### 2.6. Variant Confirmation and Segregation Analysis

To confirm the identified variants in the probands and their close relatives, we performed bidirectional Sanger sequencing. The set of primers F 5 ^′^‐ TGTGCAGGAGGTAGTGACAG ‐3 ^′^ and R 5 ^′^‐ CTCACCCCTCTCCGTTTGAT‐3 ^′^ for GCK gene, and F 5 ^′^‐ CTGGTGAGTGTCCTTGCTTG ‐3 ^′^ and R 5 ^′^‐ AGTGAGGCCATGATGAGGTT ‐3 ^′^ for HNF1A gene designed by Primer3.0 (http://bioinfo.ut.ee/primer3-0.4.0) were used. PCR for target genes was conducted using Ampliqon Master Mix (Ampliqon, Denmark) under the following parameters: initial denaturation at 95°C for 4 min, followed by 30 cycles consisting of denaturation at 95°C for 30 s, annealing at 62°C for 30 s (GCK) and 57°C for 30 s (HNF1A), elongation at 72°C for 45 s, and a final extension at 72°C for 5 min. Then, the samples were sequenced employing a Thermo Fisher 3730 DNA Analyzer and BigDye Terminator v3.1 cycle sequencing kit, and the final data were evaluated using Finch TV and Chromas software.

## 3. Results

### 3.1. WES

Then, 11 probands were considered for WES analysis to determine the exact type of diabetes. Table 1 presents the clinical characteristics of the probands.

**Table 1:** Clinical features of probands sent for WES analysis.

**Proband**	**1**	**2**	**3**	**4**	**5**	**6**	**7**	**8**	**9**	**10**	**11**
Family ID	202	201	203	204	205	101	102	103	104	105	107
Sex/age, years	F/13	F/46	M/72	F/41	F/70	F/60	F/30	M/65	F/63	F/79	F/70
Age at diagnosis, years	7	40	38	27	40	40	30	35	48	55	50
BMI, kg/m^2^	25	25	29	29.3	28.9	27	26.1	26	33.3	25.5	23.3
BMI percentile	37	37	61	64	61	50	45	39	80	40	25
Weight status	Normal weight	Normal weight	Overweight	Overweight	Overweight	Overweight	Overweight	Overweight	Overweight	Overweight	Normal weight
HbA1c, %	11.5	14	8.2	10.1	8	7.8	5	7.1	8.6	7.5	10.3
FBS	139	110	119	124	184	130	109	112	134	152	168
Total cholesterol, mg/dL	246	275	179	159	142	142	174	185	169	193	210
Treatment	Metformin	Insulin	Insulin	Insulin	Insulin	Insulin	Metformin	Insulin	Insulin	Insulin	Insulin
Complication	Neuropathy	Neuropathy	—	Neuropathy	—	Retinopathy	—	—	—	—	—
Last HbA1c, %	7.3	9.8	9.1	8.7	8.2	6.8	6.9	7.9	8.5	7.1	9.8

**Figure 1:**
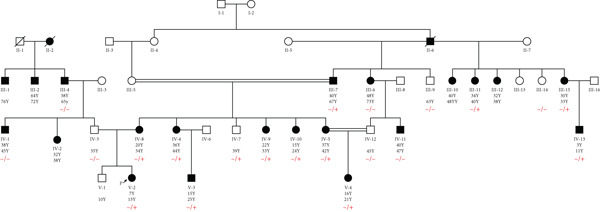
Pedigree of Family 202 tested for the GCK:c.484G > C variant. The arrow indicates the proband (V‐2). Individuals III‐4 and IV‐1 tested negative for the GCK:c.484G > C variant, indicating that the left branch of the pedigree likely has another type of diabetes. −/−: wild type; −/+: heterozygous.

**Figure 2:**
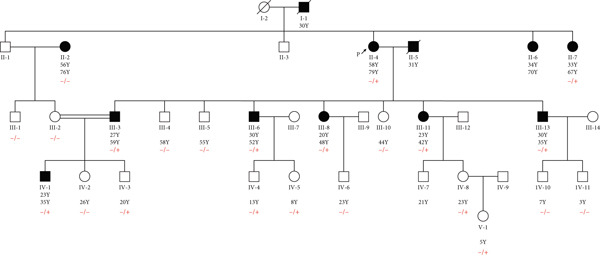
Pedigree of Family 105 tested for HNF1A:c.1136_1137del variant. The arrow indicates the proband (II‐4). The individual II‐2 tested negative for the HNF1A:c.1136_1137del variant, suggesting that the individual is probably suffering from other unknown types of diabetes. −/−: wild type; −/+: heterozygous.

Among these 11 probands, WES detected pathogenic variants related to MODY in only two cases, confirming MODY diabetes. The related pedigree for these two probands is depicted in Figures 1 and 2. One variant (HNF1A:c.1136_1137del) was previously reported as rare, while the other (GCK:c.484G > C) was novel. In Family 202, a novel heterozygous missense variant, GCK:c.484G > C(p.Gly162Arg, NM_000162.5) was identified (Figure 3a). This variant was not found in the Human Gene Mutations Database (HGMD) (http://www.hgmd.org), the gnomAD database (http://gnomad.broadinstitute.org), the 1000 Genomes Project (https://www.internationalgenome.org/), Genome Asia (https://www.genomeasia100k.org/), and Iranome (https://iranome.com/). In Family 105, an extremely rare pathogenic frameshift variant HNF1A:c.1136_1137del (p.Pro379ArgfsTer39, NM_000545.8) was identified (Figure 4a). This variant was absent in the population databases except in genomeAD, where it had an allele frequency of less than 0.001. Based on the ACMG/AMP guidelines, the GCK:c.484G > C variant was classified as likely pathogenic due to its absence in population databases (PM2), localization within a critical functional domain (PM1), presence of other pathogenic variants at the same residue (PM5), computational predictions supporting a damaging effect (PP3), and the gene′s intolerance to benign variation (PP2). The HNF1A:c.1136_1137del frameshift variant was classified as pathogenic, fulfilling criteria including predicted loss‐of‐function impact in a gene with a known LoF mechanism (PVS1), rarity in population databases (PM2), supportive segregation data (PP1), prior pathogenic reports (PP5), observed enrichment among affected individuals (PS4), and available functional data (PS3) (Table 2).

Figure 3(a) Sanger sequencing chromatograms showing the of GCK:c.484G > C(p.Gly162Arg, NM_000162.5) in Exon 5. The upper panel shows wild‐type sequence and lower represents heterozygous individuals carrying the variant. (b) AlphaFold‐predicted protein structures of GCK wild‐type and mutant (p.Gly162Arg) proteins. It shows the substitution of glycine (GLY) with arginine (ARG). The pathogenicity prediction based on AlphaMissense scores is also displayed in the wild‐type molecule (red patches indicate likely pathogenic residues). The wild type was already available for the GCK protein in AlphaFold databases; however, the mutant type was generated using the AlphaFold model in Python. (c) Heatmap from AlphaMissense. It shows pathogenicity predictions for all amino acid locations and substitutions in the GCK protein. The G162R variant is marked with a score of 0.997 (indicating strong likelihood of pathogenicity). pLDDT stands for predicted local distance difference test provided by AlphaFold.

**Figure:**
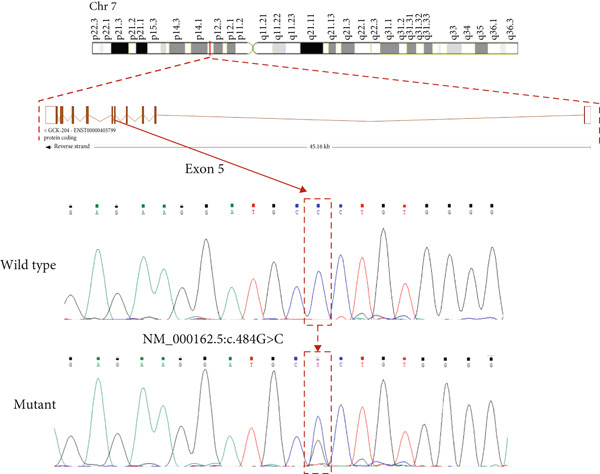
(a)

**Figure:**
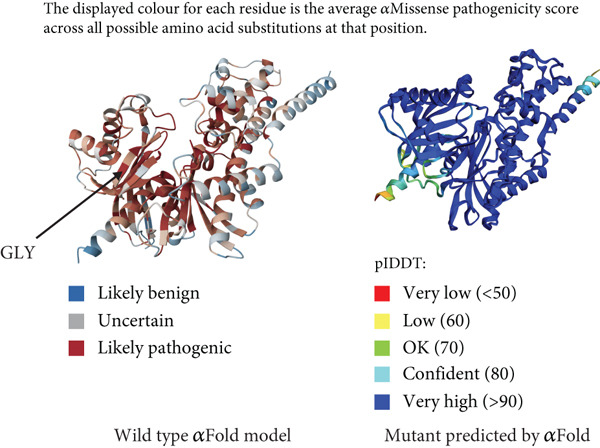
(b)

**Figure:**
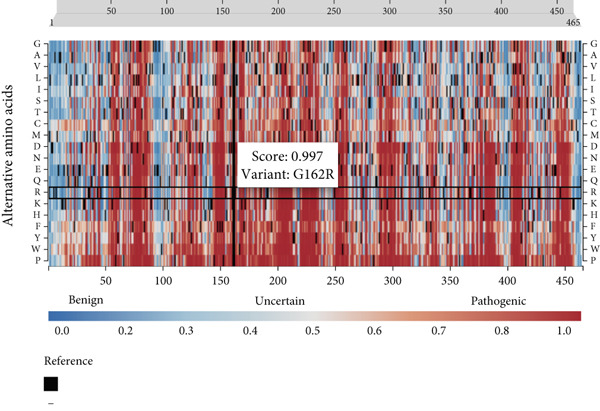
(c)

Figure 4(a) Sanger sequencing chromatograms showing the deletion of HNF1A:c.1136_1137del (p.Pro379ArgfsTer39, NM_000545.8) in Exon 6. The wild‐type sequence is shown in the upper panel, and the heterozygous deletion is shown in the lower panel. (b) AlphaFold‐predicted 3D protein structures of the HNF1A wild‐type protein. (c) Predicted protein structures of the HNF1A mutant protein (p.Pro379ArgfsTer39) demonstrating disruption of the protein′s C‐terminal region due to the frameshift variant. The wild‐type C‐terminal region displays low pLDDT scores, and the mutant structure shows a markedly shortened and disordered tail, consistent with protein truncation and functional disruption caused by the variant. pLDDT stands for predicted local distance difference test provided by AlphaFold.

**Figure:**
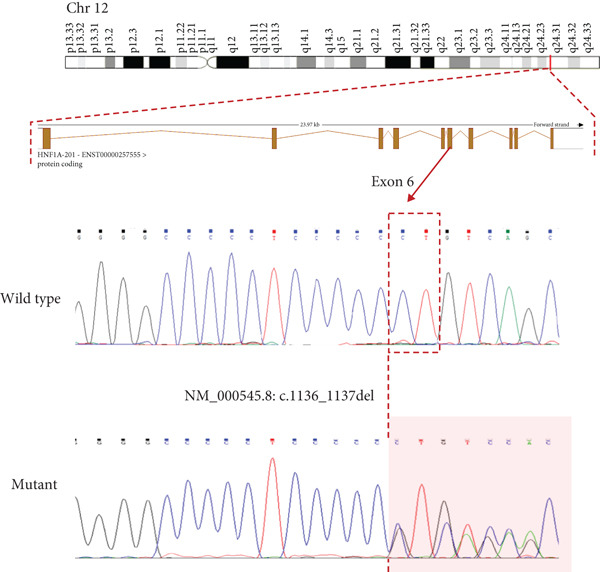
(a)

**Figure:**
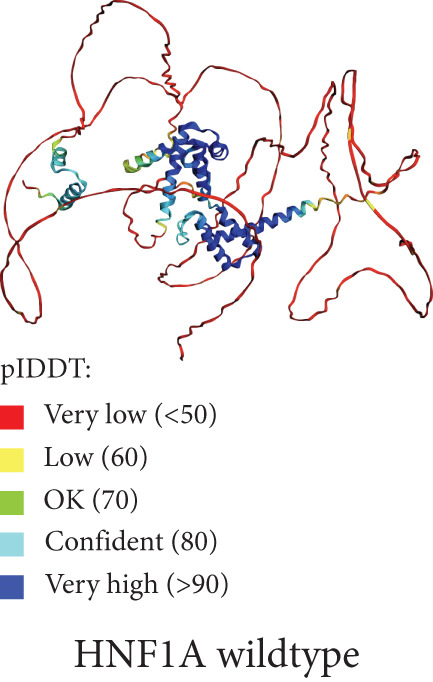
(b)

**Figure:**
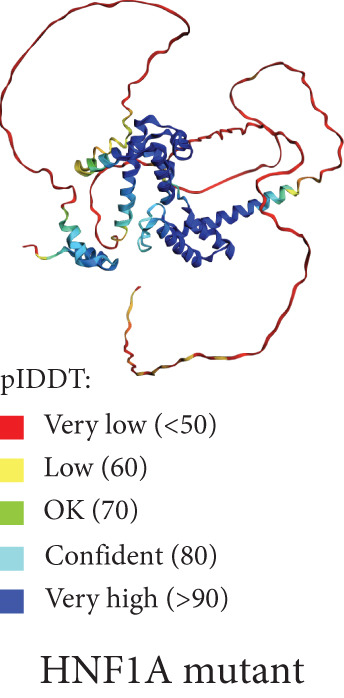
(c)

**Table 2:** Identified variants in the selected families and the results of prediction tools.

**ID**	**Chromosome coordinate**	**Gene/exon**	**Variant**	**Frequency in GenomAD/1000genome/Genome Asia/Iranome**	**Alpha missense prediction (score)**	**SIFT prediction (score)**	**MutationTaster**	**Polyphen prediction (score)**	**ACMG criteria/classification**
**#1**	Chr7: 44150064	GCK/5	NM_000162.5:c.484G > C; p.(Gly162Arg)	0/0/0/0	Deleterious(0.997)	Damaging(0.0)	Disease causing	Probably damaging(1)	PP3, PM2, PM5, PM1, PP2/likely pathogenic
**#2**	Chr12‐120996568	HNF1A/6	NM_000545.8: c.1136_1137del; p.(Pro379ArgfsTer39)	0.0001/0/0/0	N/A	N/A	Disease causing	N/A	PVS1,PM2, PS4, PS3, PP1, PP5/pathogenic

*Note:* Not applicable (N/A): as these tools are designed for missense variants. AlphaMissense prediction (score) (only for missense variants): ≥ 0.8: likely deleterious; 0.5–0.79: uncertain significance; < 0.5: likely benign; SIFT prediction (score) (only for missense variants): ≤ 0.05: damaging; > 0.05: Tolerated (benign); MutationTaster: “disease‐causing”: likely pathogenic, “polymorphism”: likely benign; PolyPhen prediction (score) (only for missense variants): 0.85–1.0: probably damaging, 0.15–0.84: possibly damaging, 0.0–0.14: benign; ACMG classification: pathogenic: strong evidence of disease association, likely pathogenic: high probability of disease association, uncertain significance: insufficient evidence for classification, likely benign/benign: unlikely to cause disease.

Abbreviations: PM1, pathogenic moderate (located in a mutational hotspot/critical domain); PM2, pathogenic moderate (absent or rare in population databases); PM5, pathogenic moderate (novel missense at a residue with other known pathogenic variants); PP1, pathogenic supporting (co‐segregation with disease); PP2, pathogenic supporting (missense variant in a gene with low benign missense variation); PP3, pathogenic supporting (multiple computational tools support a deleterious effect); PP5, pathogenic supporting (reputable source reports the variant as pathogenic); PVS1, pathogenic very strong (null variant in a gene where loss of function is a known disease mechanism); PS3, pathogenic strong (functional studies supportive of a damaging effect); PS4, pathogenic strong (increased prevalence in affected individuals.

### 3.2. In Silico Tools

Computational predictions for the GCK:c.484G > C(p.Gly162Arg, NM_000162.5) variant in Family G11 revealed deleterious by AlphaMissense (score: 0.997), damaging by SIFT (score: 0.0), and probably damaging by PolyPhen (score: 1.0), and disease‐causing by MutationTaster. According to ACMG guidelines, it was categorized as likely pathogenic. To support the pathogenicity of the GCK: p.Gly162Arg variant, we extended our analysis to incorporate additional ensemble meta‐predictors, including REVEL, CADD, MetaLR, PrimateAI, FATHMM, and DANN. All of these tools also consistently predicted a deleterious effect for the variant (Table S3) [29–34]. For the HNF1A:c.1136_1137del (p.Pro379ArgfsTer39, NM_000545.8) variant in Family 105, as a frameshift variant, AlphaMissense, SIFT, and PolyPhen were not applicable (N/A). However, MutationTaster classified it as a disease‐causing variant, and the ACMG categorized it as pathogenic (Table 2).

Figure 3b displays the AlphaFold‐predicted structure of the wild‐type and mutant protein. In addition, a heatmap of pathogenicity scores across the protein sequence with the G162R variant highlighted by a score of 0.997 (in a high‐confidence domain) was presented in Figure 3c. This score enables reliable interpretation of its structural and functional impact. Figure 4c shows the predicted structure of the mutant HNF1A, which has a markedly truncated C‐terminal region with low pLDDT scores (indicative of poor structural confidence). Although low pLDDT values are also observed in the wild‐type C‐terminal region, the mutant′s molecule shows a truncated tail, supporting its pathogenic role. AlphaFold assigns low pLDDT scores to intrinsically disordered regions (IDRs), which are common and functionally relevant in transcription factors like HNF1A. These regions lack a fixed or ordered structure, which allows them to interact with other molecules [35, 36]. The absence or shortening of IDRs can indicate functional loss in HNF1A as a transcription factor, despite structural uncertainty.

### 3.3. Variant Confirmation and Pedigree Analysis

To verify the presence of GCK:c.484G > C identified in the pedigree of 202, we performed genotyping in the proband and 21 close family members. In addition, the HNF1A:c.1136_1137del variant found in Family 105 was genotyped in the probands and 22 close relatives. The results revealed the presence of heterozygous variants in the probands. Furthermore, segregation was predominantly in accordance with the disease phenotype, although there were a few differences that could be due to age‐dependent penetrance or the presence of other kinds of diabetes (Figures 1 and 2).

## 4. Discussion

Our study employed WES to establish a genetic diagnosis of diabetes in a cohort of Iranian families in the center of Iran, Yazd. It identified pathogenic variants, including a novel GCK:c.484G > C (p.Gly162Arg) variant and a rare HNF1A:c.1136_1137del frameshift variant, confirming the MODY condition in their families. These results not only increase the mutational spectrum associated with MODY but also highlight the efficacy of WES in the correct molecular diagnosis of different types of MD.

In Yazd province where this study was done, the prevalence of diabetes is over 16% among adults, significantly above the national average of the population (10%) [37]. This increased burden of this disease in this region indicates an interaction between lifestyle variables and region‐specific genetic predispositions, and emphasizes the value of genetic identification of MD such as MODY that may be overlooked in routine clinical practice. Without region‐specific genetic data, clinicians may struggle to distinguish MODY from common diabetes types, as most MODY variant and prevalence data come from European populations [38, 39]. Two families in the current study without WES analysis might have continued to be managed as generic diabetes cases, missing the opportunity for tailored therapy. Identifying GCK‐MODY in Family 202 prevents unnecessary administration of insulin or oral hypoglycemics, thus minimizing overtreatment. In contrast, HNF1A‐MODY is characterized by progressive *β*‐cell failure, and patients harboring a pathogenic variant in this gene typically exhibit a remarkable response to low‐dose sulfonylurea medications without the need for insulin [40, 41].

HNF1A and GCK are among the most prevalent genes associated with MODY globally, although their frequency differs across populations. The predominant subtype in the United States, Japan, and Turkey is GCK‐MODY2, representing almost 50% of the identified cases [42–44]. However, HNF1A‐MODY3 is the major subtype observed in the populations of South Africa, South Korea, and Europe [45, 46]. Therefore, the detection of pathogenic variants in GCK and HNF1A genes in our population shows that the Iranian MODY profile may share traits with both Asian and worldwide patterns of subtype distribution.

In the current study, only two out of 11 investigated families were confirmed as MODY subtypes (18%). This result is comparable to a study conducted on the Kuwaiti population. This study using WES identified pathogenic variants in only seven of 31 families suspected of having MODY (22.6% positive), while the majority of the remaining families (77%) demonstrated no pathogenic variants [20]. In North Africa, a Tunisian study also revealed that the vast majority of familial early‐onset diabetes cases did not carry pathogenic variants in reported MODY genes [47]. In addition, Capan et al. investigated Turkish families with suspected MODY by WES and identified pathogenic variants in known MODY genes as well as novel candidate loci, highlighting the genetic variability of MODY and the difficulties in achieving a conclusive molecular diagnosis [48]. These results verify that known MODY genes may contribute to only a minority of cases outside of well‐studied European populations. Therefore, population‐specific studies are crucial for the genetic characterization and accurate diagnosis of MODY conditions. For instance, a population‐based genome sequencing in Qatar identified several unique variants associated with the MODY condition specific to that Middle East cohort [49]. The finding of a novel GCK variant in our study illustrates the significant allelic heterogeneity of GCK‐MODY, and the identification of a very rare HNF1A frameshift variant shows that confirmed MODY genes may also include lineage‐specific or rare variants in Iranian families.

The identification of only two pathogenic variants in recognized MODY genes is likely due to our strict inclusion criteria and filtering methodology. By considering extensive multigenerational families exhibiting clear autosomal‐dominant inheritance, our samples were enriched for classical MODY subtypes. Although our preliminary filtering was gene‐agnostic, the prioritization of rare and deleterious variants that segregated with the phenotype inherently prioritized variants in well‐established MODY genes.

The HNF1A frameshift variant (c.1136_1137del; p.Pro379fs) found in Family 105 is extremely rare according to global databases (like gnomAD) with a MAF close to zero. A limited number of MODY3 cases have previously been documented to have this particular variant, including a Chinese proband with early‐onset diabetes and autosomal dominant inheritance who displayed symptoms like progressive hyperglycemia and beta‐cell dysfunction [50]. In addition, larger European and non‐European cohorts have reported similar truncating variants in HNF1A (e.g., p.Pro291fsinsC, the most common MODY3 mutation), which frequently result in haploinsufficiency and similar clinical features, such as insulinopenia and glycosuria [51, 52]. Patients with these truncating variants usually exhibit characteristics similar to our proband, including mild‐to‐moderate hyperglycemia and initially preserved beta‐cell function [53]. Clinically, HNF1A‐MODY3 variants provide high sensitivity to low‐dose sulfonylureas, which facilitates a switch from insulin therapy and improved glycemic control [54]. In our study, the HNF1A:c.1136_1137del (p.Pro379fs) variant in Family 105 was associated with progressive hyperglycemia, consistent with the characteristic phenotype of HNF1A‐MODY [7]. In contrast, the GCK:c.484G > C (p.Gly162Arg) variant in Family 202 was associated with mild, stable fasting hyperglycemia, in accordance with the expected GCK‐MODY phenotype. However, the proband with this pathogenic variant was suffering from neuropathy, which is a rare complication in GCK‐MODY [55].

In the HNF1A‐MODY family in our study, the age of diabetes onset decreased progressively across generations (from an average of 39 in the second to 29 in the third generation), which may suggest genetic anticipation. This phenomenon is not common in MODY and remains controversial. However, a similar pattern of progressively earlier onset across generations was reported in a Japanese family with a pathogenic splice‐site variant in the HNF1B gene [56]. Therefore, this observation may warrant further investigation in the context of MD.

In the current study, while most genotype–phenotype patterns were consistent with expected autosomal‐dominant inheritance, a few individuals showed inconsistent results. These discrepancies may be ascribed to factors such as age‐dependent penetrance or potential misdiagnosis of diabetes subtype in phenotypically affected but genotype‐negative individuals. Such discrepancies are prevalent in MODY studies and demonstrate the challenges in identifying MD from other common types in large families [40].

The relatively low diagnostic yield found in our study could also be caused by a number of factors, including the limitations of WES in detecting structural variations (such as large deletions or inversions), copy number variations that contribute to MODY phenotypes but are not found in exonic regions, and noncoding regulatory variants. Additionally, uncharacterized MODY genes (often termed MODY‐X) are more prevalent in underrepresented populations, with up to 77% of Middle Eastern cases remaining unexplained [57]. Consanguinity, which is prevalent in the Middle East, including Iran (as observed in our study), can also introduce recessive or compound heterozygous variants that mimic or overlap with autosomal dominant MODY patterns, potentially making diagnosis more difficult. To enhance detection in such elusive cases, whole‐genome sequencing (WGS) with comprehensive coverage of noncoding and structural variants can be applied. In addition, trio‐WES has demonstrated higher diagnostic rates in inherited disorders and enhances the identification of de novo or inherited variants by incorporating parental data [58]. Transcriptomics, such as RNA sequencing, can also identify functional changes that DNA‐based methods miss, such as changes in expression or aberrant splicing [59], assisting in MODY‐X characterization.

While our study successfully identified MODY in two families, it also highlights several limitations. Our study focused on large, well‐defined families (an inheritance pattern across ≥ 3 generations with ≥ 7 affected individuals) to increase the chance of identifying MD with clear inheritance patterns. This strict inclusion criterion increases diagnostic yield and strengthens variant interpretation; however, it may ignore some cases with de novo pathogenic variants, smaller pedigrees, and families with incomplete penetrance. Therefore, although our findings provide valuable insights into classical MODY subtypes, they cannot be used to estimate the overall prevalence of MODY in the Iranian population. Our ability to draw broad conclusions about the MODY subtype prevalence based on only 11 families is limited. A larger sample size could potentially increase the likelihood of identifying additional subtypes (such as HNF4A‐MODY or others). In addition, although loss‐of‐function in HNF1A is a well‐established disease mechanism, functional assays were not performed on the novel *GCK* variant identified in this study, and its pathogenicity was inferred from bioinformatics and co‐segregation rather than direct laboratory evidence.

In summary, this study reports a novel missense variant (GCK: c.484G > C; p.Gly162Arg) in Family 202 and an extremely rare pathogenic frameshift variant (HNF1A:c.1136_1137del) in Family 105. Our research illustrates the effectiveness of WES in accurately determining MODY in a clinically selected cohort of Iranian families and also increases our understanding of MODY genetics. Further studies utilizing larger sample sizes, comprehensive genomic methodologies such as whole‐genome sequencing, and systematic functional validation of novel variants will be essential for identifying additional genetic etiologies and enhancing diagnostic precision, which will promote personalized diabetes management across varied populations.

## Ethics Statement

The study protocol was approved by the Ethical Committee of Shahid Sadoughi University of Medical Sciences (ID: IR.SSU.REC.1396.216), and all individuals provided informed consent.

## Disclosure

All authors read and approved the final manuscript.

## Conflicts of Interest

The authors declare no conflicts of interest.

## Author Contributions

F.S. and M.D.T. contributed equally and shared the first authorship. S.M.K. and M.Y.V.M. contributed equally, are co‐corresponding authors, and shared first authorship. F.S., M.D.T., and M.Y.V.M. conceived and designed the study. F.S., M.D.T., S.A.M., T.P., M.I., Z.S., M.R., and H.H. performed data acquisition and analysis. M.D.T. drafted the manuscript. M.R., S.M.K., and M.Y.V.M. supervised the study. S.M.K. and M.Y.V.M. provided overall project guidance. F.S., M.D.T., S.A., S.A.M., T.P., M.I., Z.S., M.R., and H.H. performed data acquisition and analysis. M.D.T. drafted the manuscript.

## Funding

No funding was received for this manuscript.

## Supporting information


**Supporting Information** Additional supporting information can be found online in the Supporting Information section. Table S1: Genotype–phenotype data in the studied families. Table S2: Summary of WES variant filtering steps for two representative families (Family 202 and Family 105). Table S3: In silico prediction scores for the GCK: p.Gly162Arg variant obtained from various ensemble and meta‐predictors.

## Data Availability

All data utilized in this work will be accessible from the corresponding author upon reasonable request.
