# Supplementary material for: Genetic Characterization of MODY in Iranian Families Using Multigenerational‐Based Whole‐Exome Sequencing Approach
Source: J Diabetes Res. 2025 Dec 30;2025:9383849. doi: 10.1155/jdr/9383849 (PMC12767221; doi:10.1155/jdr/9383849)
Supplement: Supplementary file 1 — Supporting Information Additional supporting information can be found online in the Supporting Information section. Table S1: Genotype–phenotype data in the studied families. Table S2: Summary of WES variant filtering steps for two representative families (Family 202 and Family 105). Table S3: In silico prediction scores for the GCK: p.Gly162Arg variant obtained from various ensemble and meta‐predictors. [file JDR-2025-9383849-s001.docx]

**Genetic Characterization of MODY in Iranian Families Using Multigenerational-Based Whole-Exome Sequencing Approach**

Table S1. Genotype-Phenotype data in the studied Families

| **Family ID** | **Variant** | **Individual ID** | **Age (Y)^a^** | **Diabetes Status** | **Genotype** |
| --- | --- | --- | --- | --- | --- |
| 202 | GCK:c.484G>C | III-4 | 38/65 | Affected | - / - |
| 202 | GCK:c.484G>C | III-7 | 40/67 | Affected | - / + |
| 202 | GCK:c.484G>C | III-6 | 48/73 | Affected | - / - |
| 202 | GCK:c.484G>C | III-9 | 63 | Unaffected | - / - |
| 202 | GCK:c.484G>C | III-11 | 34/40 | Affected | - / + |
| 202 | GCK:c.484G>C | III-14 | NA | Unaffected | - / - |
| 202 | GCK:c.484G>C | III-15 | 30/33 | Affected | - / + |
| 202 | GCK:c.484G>C | IV-1 | 38/45 | Affected | - / - |
| 202 | GCK:c.484G>C | IV-3 | 35 | Unaffected | - / - |
| 202 | GCK:c.484G>C | IV-8 | 20/34 | Affected | - / + |
| 202 | GCK:c.484G>C | IV-4 | 36/44 | Affected | - / + |
| 202 | GCK:c.484G>C | IV-7 | 39 | Unaffected^b^ | - / + |
| 202 | GCK:c.484G>C | IV-9 | 22/33 | Affected | - / + |
| 202 | GCK:c.484G>C | IV-10 | 15/24 | Affected | - / + |
| 202 | GCK:c.484G>C | IV-5 | 37/42 | Affected | - / + |
| 202 | GCK:c.484G>C | IV-12 | 45 | Unaffected | - / - |
| 202 | GCK:c.484G>C | IV-11 | 40/47 | Affected | - / - |
| 202 | GCK:c.484G>C | IV-13 | 3/11 | Affected | - / + |
| 202 | GCK:c.484G>C | V-2 | 7/13 | Affected | - / + |
| 202 | GCK:c.484G>C | V-3 | 15/25 | Affected | - / + |
| 202 | GCK:c.484G>C | V-4 | 16/21 | Affected | - / + |
| 105 | HNF1A:c.1136_1137del | II-2 | 56/76 | Affected | - / - |
| 105 | HNF1A:c.1136_1137del | II-4 | 58/79 | Affected | - / + |
| 105 | HNF1A:c.1136_1137del | II-7 | 33/67 | Affected | - / + |
| 105 | HNF1A:c.1136_1137del | III-1 | NA | Unaffected | - / - |
| 105 | HNF1A:c.1136_1137del | III-2 | NA | Unaffected | - / - |
| 105 | HNF1A:c.1136_1137del | III-3 | 27/59 | Affected | - / + |
| 105 | HNF1A:c.1136_1137del | III-4 | 58 | Unaffected | - / - |
| 105 | HNF1A:c.1136_1137del | III-5 | 55 | Unaffected | - / - |
| 105 | HNF1A:c.1136_1137del | III-6 | 30/52 | Affected | - / + |
| 105 | HNF1A:c.1136_1137del | III-8 | 20/48 | Affected | - / + |
| 105 | HNF1A:c.1136_1137del | III-10 | 44 | Unaffected | - / - |
| 105 | HNF1A:c.1136_1137del | III-11 | 23/42 | Affected | - / + |
| 105 | HNF1A:c.1136_1137del | III-13 | 30/35 | Affected | - / + |
| 105 | HNF1A:c.1136_1137del | IV-1 | 23/35 | Affected | - / + |
| 105 | HNF1A:c.1136_1137del | IV-2 | 26 | Unaffected | - / - |
| 105 | HNF1A:c.1136_1137del | IV-3 | 20 | Unaffected^b^ | - / + |
| 105 | HNF1A:c.1136_1137del | IV-4 | 13 | Unaffected^b^ | - / + |
| 105 | HNF1A:c.1136_1137del | IV-5 | 8 | Unaffected^b^ | - / + |
| 105 | HNF1A:c.1136_1137del | IV-6 | 23 | Unaffected | - / - |
| 105 | HNF1A:c.1136_1137del | IV-8 | 23 | Unaffected | - / + |
| 105 | HNF1A:c.1136_1137del | IV-10 | 7 | Unaffected | - / - |
| 105 | HNF1A:c.1136_1137del | IV-10 | 3 | Unaffected | - / - |
| 105 | HNF1A:c.1136_1137del | V-1 | 5 | Unaffected^b^ | - / + |

^a^: Age: onset age / current age; for unaffected individuals, current age only.

^b^: potential pre-symptomatic or reduced-penetrance cases.

Table S2: Summary of WES variant filtering steps for two representative families (Family 202 and Family 105)

| **Step** | **Description** | **Number of Variants (Family 202: GCK c.484G>C; p.Gly162Arg)** | **Number of Variants (Family 105: HNF1A c.1136_1137del)** |
| --- | --- | --- | --- |
| 1 | All called recalibrated variants | 72338 | 73587 |
| 2 | Annotation by ANNOVAR | 63923 | 65395 |
| 3 | Heterozygous variants | 21467 | 24371 |
| 4 | Exclusion of >0.01 allele frequency | 1872 | 2184 |
| 5 | Functional variants (Missense, Nonsense, Frameshift, splice-site, Start-loss/stop-gain | 512 | 583 |
| 6 | Exclusion of Benign, Synonymous, & Intergenic variants | 308 | 336 |
| 7 | Selection of variants in the phenotype-related genes | 36 | 23 |
| 8 | Variant filtering based on clinical significance and in silico pathogenicity prediction | 1 | 1 |

| **ID** | **Chromosome coordinate** | **Gene/Exon** | **Variant** | **REVEL** | **BayesDel(noAF)** | **MetaLR** | **PrimateAI** | **FATHMM** | **DANN** |
| --- | --- | --- | --- | --- | --- | --- | --- | --- | --- |
| **#1** | chr7: 44150064 | GCK/5 | NM_000162.5:c.484G>C; p.(Gly162Arg) | Deleterious (Strong) (0.99) | Deleterious (Strong) (0.54)) | Deleterious (0.99) | Deleterious (Moderate) (0.93) | Deleterious (Moderate) (-6.53) | Deleterious (1) |

Table S3. In silico prediction scores for the GCK:p.Gly162Arg variant obtained from various ensemble and meta-predictors

Note: The tools REVEL, BayesDel, MetaLR, PrimateAI, FATHMM, and DANN are designed for missense variants only and therefore do not provide scores for frameshift variants such as HNF1A:c.1136_1137del.
